# Design of a Carrier Wave for Capacitive Transducer with Large Dynamic Range

**DOI:** 10.3390/s20040992

**Published:** 2020-02-12

**Authors:** Zhu Li, Xian Zhang, Shu Zou, Xiangqing Huang, Chao Xue, Jianping Liu, Qi Liu, Shanqing Yang, Liangcheng Tu

**Affiliations:** TianQin Research Center for Gravitational Physics and School of Physics and Astronomy, Sun Yat-sen University (Zhuhai Campus), Zhuhai 519082, China; lizhu@mail.sysu.edu.cn (Z.L.); zhangx675@mail2.sysu.edu.cn (X.Z.); zoush6@mail2.sysu.edu.cn (S.Z.); xuech7@mail.sysu.edu.cn (C.X.); liujp39@mail.sysu.edu.cn (J.L.); liuq239@mail.sysu.edu.cn (Q.L.); yshq@mail.sysu.edu.cn (S.Y.); tuliangch@mail.sysu.edu.cn (L.T.)

**Keywords:** capacitive transducer, carrier wave, dynamic range, displacement measurement

## Abstract

Capacitive transducers are widely used in fundamental physics experiments, seismology, Earth or planetary observations, and space scientific and technical applications because of their high precision, simple structure, and compatibility with various measurements. However, in real applications, there is a trade-off between their resolution and dynamic range. Therefore, this paper is aimed at enlarging the dynamic range while ensuring high resolution. In this paper, a noise analysis of a capacitive transducer is presented, which shows that the amplitude noise of the carrier wave is the main limiting factor. Hence, a new method of generating a carrier wave with lower-amplitude noise is proposed in the paper. Based on the experimental verification, it is found that the carrier wave produced through the new method performed significantly better than the typical digital carrier wave when they were compared in the same sensing circuit. With the carrier wave produced through the new method, the dynamic range of the capacitive transducer can reach 120.7 dB, which is 18.3 dB greater than for the typical direct digital synthesis (DDS) method. In addition, the resolution of the carrier wave is mainly limited by the voltage reference components.

## 1. Introduction

Capacitive position transducers have many advantages such as high precision, a simple structure, and compatibility with various measurements [1,2]. Thus, they are widely used in fundamental physics experiments and space scientific and technical applications [3,4,5]. Fundamental physics experiments such as tests of equivalent principle [6,7], research on new interactions, or the direct observation of gravity waves [8,9,10,11] require the measurement of extremely weak acceleration. The application field is so soft that a drag-free compensation system is operated by a dedicated satellite. However, a higher accuracy results in a smaller dynamic range. Thus, the trade-off between the dynamic range and the sensitivity of the accelerometer is under consideration in numerous applications, such as natural disaster monitoring, gravity field measurement, mineral resources exploration, and geophysical inspection for mineral products [12,13]. In the field of airborne gravity gradient measurement, ultra-sensitive and large-dynamic-range accelerometers are applied for the measurement of the variation of gradient signals in a gravitational field [14]. Accelerometers that are employed in a GGI (gravitational gradient instrument) have a resolution of 0.01 ng/√Hz at 1 Hz (1 g ≈ 9.8 m/s^2^) and a large dynamic range that enable them to work properly [15]. Therefore, a high-precision accelerometer with a larger dynamic range has important application prospects.

A capacitive position transducer uses two fixed capacitive plates mounted on a frame and a moving capacitive plate to form a differential capacitive pair. Once there is motion of the frame, the gap between the moving plate and the two fixed plates varies. Then, differential capacitance appears. To reduce the low-frequency noise and convert the signal to an easily measurable voltage, carrier waves with opposite phases are imposed on the two fixed plates. Obviously, the noise of the carrier wave directly determines the noise floor of the capacitive transducer.

In this paper, the principle of a capacitive transducer is firstly introduced. Secondly, the noise of the capacitive transducer is analyzed, indicating that the dominant noise is contributed by the carrier wave. Subsequently, a new method for carrier wave generation is demonstrated in detail. Finally, an experiment is carried out to verify the performance of the new carrier wave.

## 2. Principle of Capacitive Transducers

**Figure 1:**
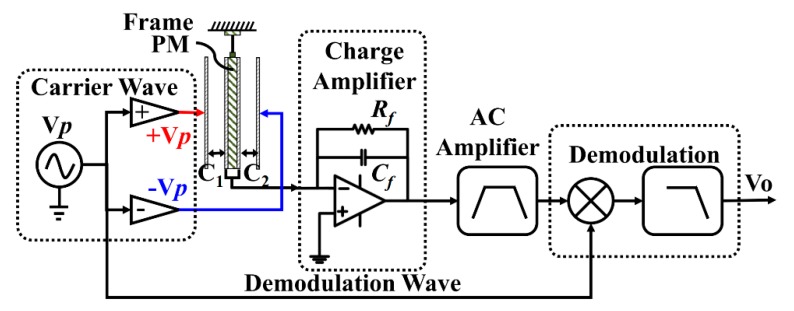
Diagram of a typical capacitive transducer, including carrier wave modulation, charge amplifier, alternating current (AC) amplifier, and demodulation. *V_p_* is the carrier wave and also the demodulation wave. *C_1_* and *C_2_* are the differential capacitances. In a charge amplifier, *R_f_* is the feedback resistance and *C_f_* is the feedback capacitance.

A diagram of a typical capacitive transducer is shown in Figure 1, including carrier wave modulation, charge amplifier, alternating current (AC) amplifier, and demodulation [16]. *V_p_* is a carrier wave to modulate the capacitance variation signal, which is generally a high-frequency sine wave or square wave. For a capacitive sensing accelerometer, differential electrodes can be designed to eliminate the common mode noise. As a moving plate, both sides of the proof mass (PM) are coated with gold. The PM and the left-side and right-side fixed capacitance plates form a pair of differential measurement capacitances, *C*_1_ and *C*_2_; they are equal when the PM stays at the balance position. Once an external acceleration is applied to the frame, there is a movement of the PM. Thus, a capacitance difference Δ*C* = *C*_1_ − *C*_2_ is formed, which indicates the external acceleration. A pair of carrier waves, which are +*V_p_* and −*V_p_* with equal amplitude and opposite phase, are injected into the two fixed plates. Therefore, the differential capacitance Δ*C* information is converted to voltage information and picked up by the charge amplified by *V_charge_* [17].

Charge amplifiers are widely used in capacitive transducers, for which a schematic diagram is shown in Figure 1. *C_f_* is the feedback capacitance, which determines the gain of the charge amplifier. *R_f_* is the feedback resistance, which is used to avoid outrange in direct current (DC). The output of the charge amplifier *V_charge_* can be written as

(1) $$V_{charge}=V_{p}\frac{sR_{f}\Delta C}{1+sR_{f}C_{f}},$$

where *s* is the Laplace variable. Normally *sR_f_ C_f_* ≫ 1; thus, Equation (1) can be simplified to the following equation:

(2) $$V_{charge}=V_{p}\frac{\Delta C}{C_{f}}.$$

In our design, the amplitude of *V_p_* was 5 V and *C_f_* was 1 pF. Thus, the gain of the charge amplifier was 5 V/pF. The maximum value of Δ*C* was 2 pF.

Similarly, the gains of the AC amplifier and demodulation unit are shown in Table 1. It is worth noting that the demodulation unit included a multiplier and a low-pass filter.

The terminal output voltage signal of the capacitive transducer can be expressed as follows:

(3) $$V_{out}=V_{p}\cdot \frac{\Delta C}{C_{f}}\cdot G_{BP}\cdot G_{M}\cdot G_{LP}.$$

## 3. Noise Analysis and Design of Large-Dynamic-Range Carrier Wave

### 3.1. Noise Analysis of the Capacitive Transducer

Upon separating Equation (3) into three parts (*V_p_*, Δ*C*, and the gain of the rest *G*_T_), it can be simplified as follows:

(4) $$V_{out}=V_{p}\cdot \Delta C\cdot G_{T},$$

where $G_{T}=G_{BP}\cdot G_{M}\cdot G_{LP}/C_{f}$.

Considering the intrinsic background noise *V_bgn_* of the circuit caused by amplifiers, resistances, and other devices, the final noise equation of the output voltage can be given by

(5) $$V_{\mathrm{Out},n}^{2}={\left(\Delta C\cdot G_{T}\cdot \delta V_{p}\right)}^{2}+{\left(V_{p}\cdot \Delta C\cdot \delta G_{T}\right)}^{2}+{\left(V_{p}\cdot G_{T}\cdot \delta \Delta C\right)}^{2}+V_{bgn}^{2},$$

where *δV_p_* is the noise caused by the carrier wave, *δG*_T_ is the instability of the transducer circuit gain, and *δ*Δ*C* is the disturbance of the signal, which limits the best resolution [18,19,20,21]. Actually, many factors can influence the *δ*Δ*C*, such as temperature, humidity, air pressure, external environment vibration, and so on. Thus, both the design and the environment determine the δΔ*C*. Therefore, in order to get better resolution, it is effective to make the deterioration influence of *δV_p_* and *δG*_T_ less than that of *δ*Δ*C*. Similarly, the noise influence of *V_bgn_* should be as small as possible.

In this article, a capacitive transducer with a large dynamic range is expected. Here, the dynamic range (*DR*) is defined as

(6) $$DR=-10\cdot \mathrm{lg}\frac{V_{\mathrm{Out},n}^{2}}{V_{\mathrm{Out},\mathrm{MAX}}^{2}}=-10\cdot \mathrm{lg}\left[{\left(\frac{\delta V_{p}}{V_{p}}\right)}^{2}+{\left(\frac{\delta G_{T}}{G_{T}}\right)}^{2}+{\left(\frac{V_{bgn}}{V_{\mathrm{Out},\mathrm{MAX}}}\right)}^{2}+{\left(\frac{\delta \Delta C_{\mathrm{MAX}}}{\Delta C}\right)}^{2}\right],$$

where *V*_Out,MAX_ and Δ*C*_MAX_ are the full-scale voltage and differential capacity, respectively. Obviously, the dynamic range of the capacitive transducer is closely related to the carrier wave’s dynamic range, the gain stability, and the background noise of circuit. Next, the factors are analyzed separately.

Firstly, we investigate the gain instability *δG*_T_, which is ignored by many articles, and believed to result from environmental noise. In this article, a brief estimation of the noise effect of *δG*_T_ is given. Since the resistance and capacitance determine the gain of the circuit, and temperature has the main influence on resistance and capacitance, the effect of temperature needs to be considered. However, in the bandwidth we consider (0.1–10 Hz), the circuit was measured in our cave lab, where the disturbance of temperature was lower than 10^−5^ K/Hz^1/2^ [12,13]. As the temperature coefficient of the resistance and the capacitance was about 100 ppm/K, the gain was stable enough in the concerned bandwidth. Therefore, the gain instability could not be the major limiting factor of the dynamic range.

**Figure 2:**
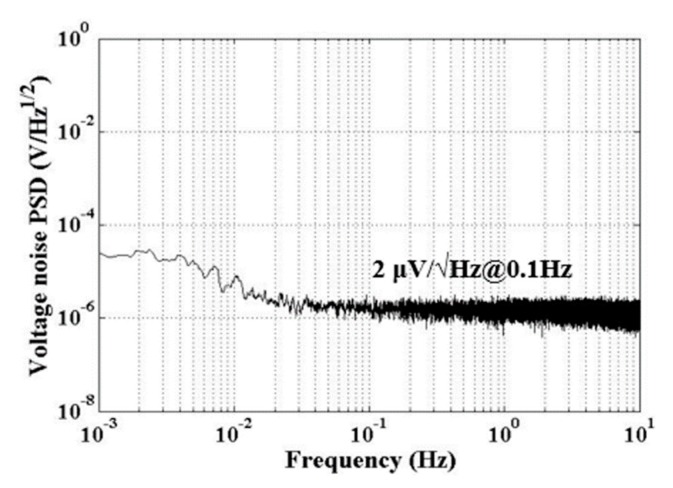
The background noise of capacitive transducer. It is about 2 μV/Hz^1/2^ at 0.1 Hz. The noise is consistent with the theoretical calculation in Table 2.

Secondly, the intrinsic background noise also limits the dynamic range. The background noise could be theoretically calculated and validated by experiments. The results are shown in Table 2 and Figure 2. The total background noise of the capacitive transducer was about 2 μV/Hz^1/2^ in the concerned bandwidth, which was consistent with the theoretical calculation. Thus, the intrinsic background noise limited the dynamic range to be narrower than 134 dB due to the maximum output being 12 V.

Finally, the carrier waves are taken as a voltage source, whose dynamic range is the direct limit of the capacitive transducer’s dynamic range. The signal wave and the demodulation wave can be written as follows:

(7) $$V_{sig}=A\cdot \sin\left(2\pi f_{s}t+\varphi_{s}\right),$$

(8) $$V_{dem}=\sin\left(2\pi f_{s}t\right),$$

where *A* and *f_s_* are the amplitude and frequency of the carrier wave, respectively. As Figure 1 shows, the demodulation wave and signal wave are homologous; thus, the frequency of the two waves are the same. *φ_s_* is the phase difference between the signal wave and the demodulation wave. Both the amplitude and the phase influence the carrier wave’s noise.

Supposing that the phase disturbance is *δφ_s_*; then, the output and the phase noise after demodulation can be written as in Equations (9) and (10).

(9) $$V_{p,\varphi_{s}}=\frac{A}{2}\cdot \cos\left(\varphi_{s}\right),$$

(10) $$V_{p,\varphi_{s},n}=\frac{A}{2}\cdot \sin\left(\varphi_{s}\right)\cdot \delta \varphi_{s}\approx \frac{A}{2}\cdot \varphi_{s}\cdot \delta \varphi_{s}.$$

As Equations (9) and (10) reveal, the phase difference *φ_s_* should be kept to zero during demodulation; generally, the *φ_s_* can easily be controlled within 0.1 degree (~1.7 mrad). The resistance, capacitance, and environmental noise are the main causes of the phase noise *δφ_s_*. As with the gain stability, the phase stability would be better than 160 dB, which is not the main concern of the DR.

In Equation (6), it can be seen that the amplitude noise of the carrier wave also limits the dynamic range. Generally, the stability of voltage magnitude would be 100–120 dB in our concerned frequency range, which makes it the key constraint on the dynamic range.

In summary, the dynamic range of the capacitive transducer is limited by the amplitude stability of a carrier wave in the concerned bandwidth. How to design a stable carrier wave for the large-dynamic-range circuit is the key.

### 3.2. Design of Large-Dynamic-Range Carrier Wave

**Figure 3:**
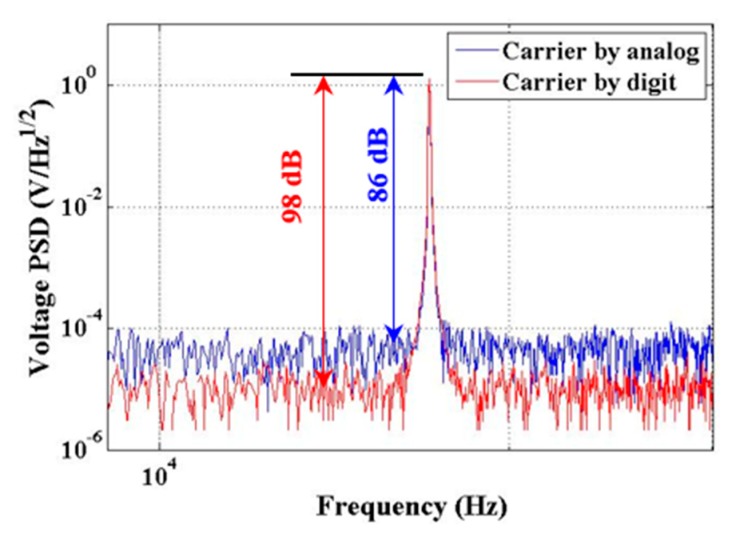
The contrast between the analog and digital methods of producing a carrier wave. The red line is the digital method, which has a better performance in the center carrier frequency.

There are many methods for producing carrier waves, which can be categorized as analog circuits or digital circuits. The digital circuit of a carrier wave consists of digital control chips (FPGA, which means field-programmable gate array, or DSP, which means digital signal processing), a digital-analog converter (DAC), a filter, and so on. The digital carrier wave in this article was generated by direct digital synthesis (DDS), and output as analog voltage through the DAC. The precision of DAC determines the performance of the carrier wave. The self-oscillation circuit and fixing amplitude circuit can produce a 100-kHz carrier wave via the traditional analog method. The frequency stability of the carrier wave depends on the resistance and capacitance of the frequency selection, which makes it worse than the digital method. The amplitude of the carrier wave is restricted by a nonlinear component whose gain varies. Thus, it is difficult to get a more stable carrier wave than that produced by the digital method. In Figure 3, the contrast between the analog and digital methods is shown.

**Figure 4:**
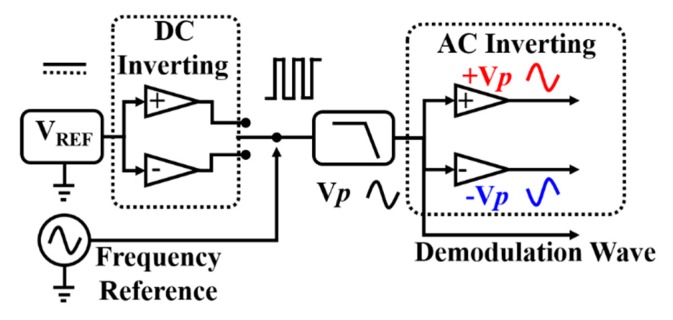
The block diagram of carrier wave production, consisting of voltage reference, switch, filter, inverting circuit, and frequency reference.

The DDS realization of the carrier wave shows higher resolution than the traditional analog realization (the self-oscillation circuit). Moreover, there could be higher precision and more stable digital carrier waves, but this is not common due to the very high cost and high complexity. Here, we propose a new analog design for a carrier wave with higher precision and frequency stability, which performs better than the typical DDS method and is easy to operate and integrate. The idea is to separate the frequency and the amplitude first, then use high-precision sources to supply the stable amplitude and frequency, and finally get our carrier wave by filtering. The amplitude of the carrier wave is affected only by the voltage reference, which is not limited by the bits of the DAC. The structure of the design is shown in Figure 4, which includes the voltage reference, frequency reference, switch, filter, and inverse circuit.

In Figure 4, the voltage source is a stable DC voltage. We can get two opposite voltage sources via the inverting circuit. The reference frequency wave (100-kHz sine or square wave) controls the switch to choose a positive port or negative port. Thus, we obtain a square wave at 100 kHz. Subsequently, a low-pass filter shaped the square wave to be a sine wave. The noise of the amplitude is limited by the voltage reference stability only. However, the noise of the inverting circuit and filter also need to be evaluated. The evaluation results are shown in Table 3; generally, the noise level of the voltage reference was about μV/Hz^1/2^, which indicates that the voltage reference is the main limit of the amplitude noise of the carrier wave. Relative to the traditional method of producing a carrier wave, the new method can improve the dynamic range. In addition, the performance of the voltage source is closely related to the dynamic range of the capacitive transducer.

## 4. Experimental Results

In order to verify whether the carrier wave is the main factor that limits the dynamic range of the transducer, two constant capacitances were selected to form a pair of differential capacitances for the transducer. As analyzed before, the carrier wave noise (*δA*) and the stability of the gain (*δG*_T_) were associated with Δ*C*. However, the *δG*_T_ was verified; thus, it could be ignored. Thus, we could analyze the carrier wave’s noise *δA* by inputting a different Δ*C*. Then, the carrier waves generated by the new method and the typical DDS method were tested and compared in the same sensing circuit. Finally, we verified that the amplitude noise of the carrier was the main source of the noise of the capacitive transducer.

**Figure 5:**
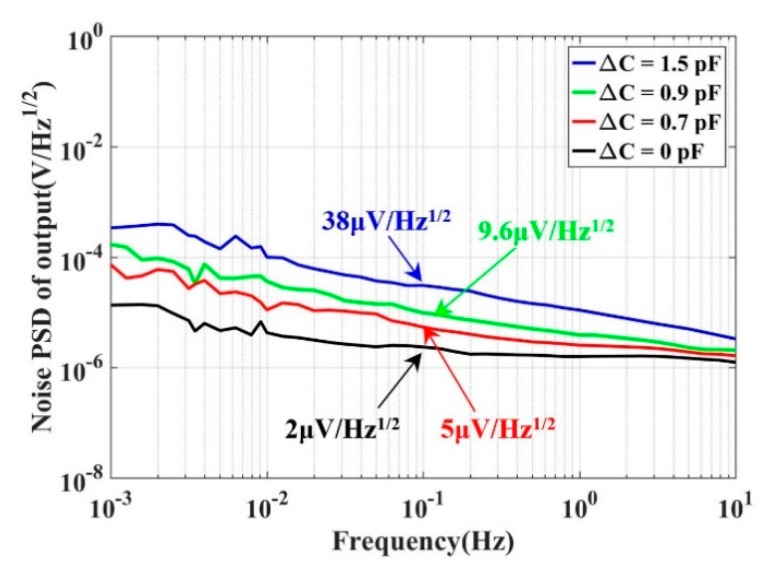
The output noise of the capacitive transducer with different Δ*C* values. This indicates that a larger differential capacitance Δ*C* results in a worse output noise.

The two constant capacitances had stable performance, with a difference of *δ*Δ*C* ≈ 0. Therefore, we could evaluate the noise of the signal wave by experiments. The power spectral density (PSD) of the capacitive transducer output is shown in Figure 5. Four different constant capacitance combinations (*C*_1_, *C*_2_) were used: *C*_1_ = *C*_2_ = 4.7 pF, Δ*C* = 0 pF; *C*_1_ = 4.3 pF, *C*_2_ = 5 pF, Δ*C* = 0.7 pF; *C*_1_ = 4.1 pF, *C*_2_ = 5 pF, Δ*C* = 0.9 pF; and *C*_1_ = 4 pF, *C*_2_ = 5.5 pF, Δ*C* = 1.5 pF. Moreover, the capacitances of *C*_1_ and *C*_2_ were evaluated by a high-precision LCR meter. The carrier waves were produced using the DDS method. The amplitude and frequency were 2.5 V and 100 kHz, respectively.

A larger differential capacitance Δ*C* resulted in a worse output noise at 0.1 Hz. Meanwhile, the noise was higher than the background noise, as shown in Figure 2. As a result, we can preliminarily conclude that the output noise of the capacitive transducer was mainly from the carrier wave in our concerned frequency.

**Figure 6:**
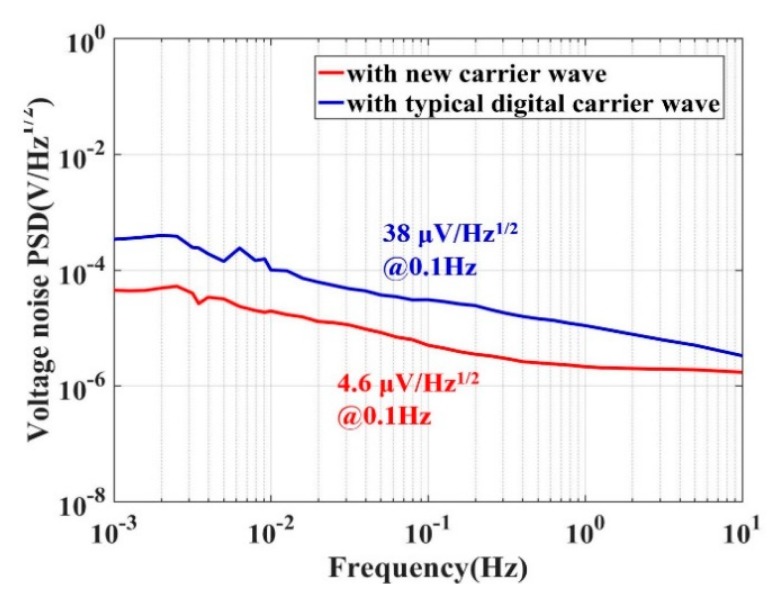
A comparison of capacitive transducers, with different carrier waves, allowing the output to reach the maximum value of 5 V. The red line represents the new carrier wave, while the blue one represents the typical direct digital synthesis (DDS) carrier wave.

Then, the new design of the carrier wave implementation was tested and compared with the typical digital carrier wave (generated by DDS, and output analog voltage through DAC), which was proven to have good performance among the carrier waves generated by other traditional methods. AD580, used for voltage reference, has a high-precision voltage reference of 2.5 V. The frequency reference is a 100-kHz crystal oscillator. AD630 is used for DC inverse and switch, which is a balance modulator/demodulator with 2-MHz bandwidth. The cut-off frequency of the low-pass filter is 120 kHz. Only the carrier wave generator is replaced in the capacitive transducer. For better contrast, the output of each circuit is set to a maximum of 5 V, which corresponds to an input differential capacitive (Δ*C*) of about 1.5 pF. Figure 6 shows the noise PSDs of the capacitive transducer with different carrier waves. The results show that the PSD of the noise with the typical DDS carrier wave was 38 μV/√Hz, which corresponds to a dynamic range of 102.4 dB. By using the new carrier wave, the PSD of the noise decreased to 4.6 μV/√Hz, corresponding to a dynamic range of 120.7 dB.

**Figure 7:**
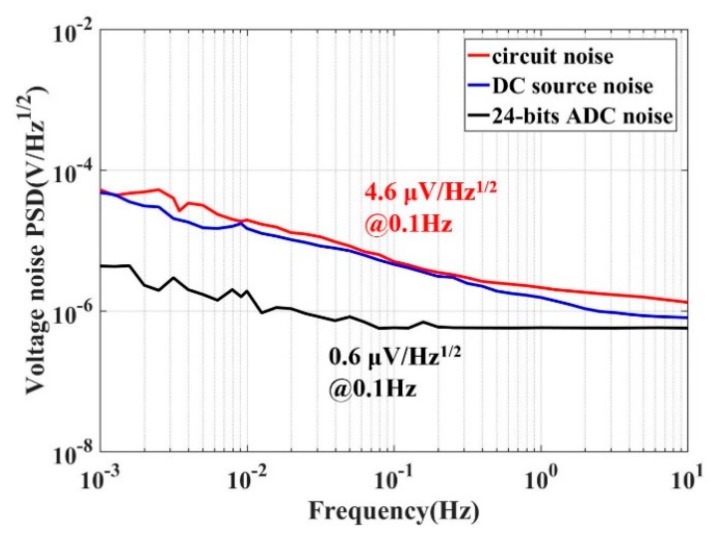
Comparing the noise of the voltage reference and the capacitive transducer at 0.1 Hz with the same maximum output of 5 V. The red line comes from the capacitive transducer and the blue one is caused by the DC source being kept constant at 4.6 μV/Hz^1/2^ at 0.1 Hz. The black line is the background noise of the 24-bit analog-digital converter (ADC).

Next, we verified that the noise source of the capacitive transducer was the amplitude noise of the carrier wave. The PSDs of the reference voltage, the output of the capacitive transducer, and the intrinsic noise of the analog-digital converter (ADC) are shown in Figure 7. The results showed that the PSD of the transducer and the PSD of the reference voltage were close to 4.6 μV/√Hz at 0.1 Hz and much bigger than that of the ADC, which indicates that the noise and dynamic range was limited by the source performance in the case of full-scale output.

## 5. Conclusions and Future Prospects

In this project, a typical capacitive transducer was used. The amplitude noise of the carrier wave was proven to be the major factor that determined the performance of the capacitive transducer. A new method to produce a carrier wave was designed. The new carrier wave is not only easily operated and integrated, but also has a large dynamic range. The amplitude noise of the new carrier wave is limited only by the stability of the voltage source, unlike the digital carrier wave’s, which is limited by the bits of DAC. Therefore, with the new method, it is easier to get lower noise and a larger dynamic range than with the digital method. In the case of full scale, keeping the same gains of capacitive transducer, a typical DDS carrier wave had a noise of 38 μV/Hz^1/2^ with a dynamic range of 102.4 dB, but the new carrier wave reached a noise value of 4.6 μV/Hz^1/2^ with a dynamic range of 120.7 dB. The design obtained a dynamic range 18.3 dB larger than that of the typical DDS method. The performance of the carrier wave generated through the new method was limited by the voltage reference components.

In order to further improve the dynamic range of the capacitive transducer in the concerned frequency, a voltage reference with better performance is the key. This paper aimed to improve the dynamic range; the sensitivity of the total capacitive transducer was 2.54 V/pF. If the sensitivity changes, the background noise of circuit may have a major influence, and the dynamic range could decrease. In the sensors, the design of mechanical parts can offset the sensitivity to acceleration.

## Figures and Tables

**Table 1:** The gains of each part in the capacitive transducer.

Circuit Units	Gain
Charge amplifier	5 V/pF
AC amplifier (*G_AC_*)	0.4
Multiplier (*G_M_*)	4/π
Low-pass filter (*G_LP_*)	1

**Table 2:** The noise of each part, converted to the end of the capacitive transducer by theoretical calculation.

Circuit Units	Noise at the End of Circuit
Charge amplifier	76 nV/Hz^1/2^
AC amplifier	189 nV/Hz^1/2^
Multiplier	2 μV/Hz^1/2^
Low-pass filter	200 nV/Hz^1/2^
Total noise	2.02 μV/Hz^1/2^

**Table 3:** The noise of the circuit units for carrier wave production. DC—direct current.

Circuit Units	Noise at the End of Circuit
Voltage reference	Limited by components
DC inverting circuit	200 nV/Hz^1/2^
Filter	27 nV/Hz^1/2^
AC inverting circuit	15 nV/Hz^1/2^

## References

[1] Jones R.V., Richards J. (1973). The design and some applications of sensitive capacitance micrometers. J. Phys. E Sci. Instrum..

[2] Baxter L.K., Herrick R.J. (1997). Capacitive Sensors: Design and Applications.

[3] Touboul P., Foulon B., Willemenot E. (1999). Electrostatic space accelerometers for present and future missions. Acta Astronaut..

[4] Touboul P., Willemenot E. (1999). Accelerometers for CHAMP, GRACE and GOCE space missions: Synergy and evolution. Bollett. Geofis. Teor. Appl..

[5] Josselin V., Touboul P., Kielbasa R. (1999). Capacitive detection scheme for space accelerometers applications. Sens. Actuators A.

[6] Sumner T.J., Anderson J., Blaser J.P., Cruise A.M., Damour T., Dittus H., Everitt C.W.F., Foulon B., Jafry Y., Kent B.J. (2007). STEP (satellite test of the equivalence principle). Adv. Space Res..

[7] Zhou Z.B., Dong Y.J., Tian Y.L., Qin D., Luo J. (2004). Excitation frequency effect of differential capacitance transducer for equivalence principle test. Chin. Phys. Lett..

[8] Cavalleri A., Dolesi R., Fontana G. (2001). Progress in the development of a position sensor for LISA drag-free control. Class. Quantum Grav..

[9] Weber W.J., Cavalleri A., Dolesi R., Fontana G., Vitale S. (2002). Position sensors for LISA drag-free control. Class. Quantum Grav..

[10] Gan L., Mance D., Zweifel P. (2012). LTP is FEE sensing channel: Front-end modeling and symmetry adjustment method. IEEE Sens. J..

[11] Weber W.J., Bortoluzzi D., Carbone L., da Lio M., Dolesi R. (2003). Position sensors for flight testing of LISA drag-free control. Proc. SPIE-ISOP.

[12] Yan S.T., Xie Y.F., Deng Z.G. (2017). A subnano-g electrostatic force-rebalanced flexure accelerometer for gravity gradient instruments. Sensors.

[13] Wu W.J., Liu J.Q., Tu L.C. (2018). A nano-g micromachined seismic sensor for levelling-free measurements. Sens. Actuators A Phys..

[14] Lee J.B. (2001). FALCON gravity gradiometer technology. Explor. Geophys..

[15] Metzger E.H. (1977). Recent gravity gradiometer developments. Am. Inst. Astronaut. Aeronaut..

[16] Huang X.Q., Deng Z.G., Xie Y.F. (2017). A new scale factor adjustment method for magnetic force feedback accelerometer. Sensors.

[17] Armano M. (2017). Capacitive sensing of test mass motion with nanometer precision over millimeter-wide sensing gaps for space-borne gravitational reference sensors. Phys. Rev. D.

[18] Hu M., Bai Y.Z., Zhou Z.B., Li Z.X., Luo J. (2014). Resonant frequency detection and adjustment method for a capacitive transducer with differential transformer bridge. Rev. Sci. Instrum..

[19] Wu W.J., Liu D.D., Tu L.C. (2018). A precise spacing-control method in MEMS packaging for capacitive accelerometer applications. J. Micromech. Microeng..

[20] Bertolini A., DeSalvo R., Fidecaro F., Francesconi M., Marka K., Sannibale V. (2006). Readout system and predicted performance of a low-noise low-frequency horizontal accelerometer. Nucl. Instrum. Methods Phys. Res. A.

[21] Bai Y.Z., Zhou Z.B., Tu H.B. (2009). Capacitive position measurement for high-precision space inertial sensor. Front. Phys. China.

